# Development of Novel Peptides for the Antimicrobial Combination Therapy against Carbapenem-Resistant *Acinetobacter baumannii* Infection

**DOI:** 10.3390/pharmaceutics13111800

**Published:** 2021-10-27

**Authors:** Joonhyeok Choi, Ahjin Jang, Young Kyung Yoon, Yangmee Kim

**Affiliations:** 1 Department of Bioscience and Biotechnology, Konkuk University, Seoul 05029, Korea; jun9688@konkuk.ac.kr (J.C.); ajin931017@konkuk.ac.kr (A.J.); 2 Division of Infectious Diseases, Department of Internal Medicine, Korea University College of Medicine, Seoul 02841, Korea; young7912@korea.ac.kr

**Keywords:** antimicrobial peptides, antibiotics, synergistic effect, CRAB

## Abstract

Carbapenem-resistant *Acinetobacter baumannii* (CRAB) infection has a high mortality rate, making the development of novel effective antibiotic therapeutic strategies highly critical. Antimicrobial peptides can outperform conventional antibiotics regarding drug resistance and broad-spectrum activity. PapMA, an 18-residue hybrid peptide, containing N-terminal residues of papiliocin and magainin 2, has previously demonstrated potent antibacterial activity. In this study, PapMA analogs were designed by substituting Ala^15^ or Phe^18^ with Ala, Phe, and Trp. PapMA-3 with Trp^18^ showed the highest bacterial selectivity against CRAB, alongside low cytotoxicity. Biophysical studies revealed that PapMA-3 permeabilizes CRAB membrane via strong binding to LPS. To reduce toxicity via reduced antibiotic doses, while preventing the emergence of multi-drug resistant bacteria, the efficacy of PapMA-3 in combination with six selected antibiotics was evaluated against clinical CRAB isolates (C1–C5). At 25% of the minimum inhibition concentration, PapMA-3 partially depolarized the CRAB membrane and caused sufficient morphological changes, facilitating the entry of antibiotics into the bacterial cell. Combining PapMA-3 with rifampin significantly and synergistically inhibited CRAB C4 (FICI = 0.13). Meanwhile, combining PapMA-3 with vancomycin or erythromycin, both potent against Gram-positive bacteria, demonstrated remarkable synergistic antibiofilm activity against Gram-negative CRAB. This study could aid in the development of combination therapeutic approaches against CRAB.

## 1. Introduction

The emergence of multi-drug resistant (MDR) bacteria, combined with the failure of most antibiotic candidates in clinical trials, poses a serious threat to global public health [1,2,3]. In particular, diseases caused by Gram-negative bacteria, such as postoperative wound infection, urinary tract infection, hospital-acquired pneumonia, catheter-associated bloodstream infection, meningitis, and sepsis [4,5], have high mortality. Carbapenems such as doripenem, imipenem, and meropenem are generally considered to be the final choice of treatment for MDR Gram-negative bacteria; however, these bacteria have recently begun to show increased resistance to these drugs. MDR bacterial infections featuring carbapenem-resistant *Acinetobacter baumannii* (CRAB) are at the top of the World Health Organization (WHO) priority list for the development of new antibiotics [6,7,8]. Therefore, there is a need to accelerate the development of new antibiotic therapeutic strategies.

As antibiotic resistance develops rapidly after the introduction of new antimicrobial agents, it is necessary to develop antimicrobial compounds with novel mechanisms that differ from those of conventional antibiotics. Antimicrobial peptides (AMPs) are diverse, and they are produced by various living organisms [9,10], where they are known to participate in the organism’s innate immunity [11,12,13]. Unlike conventional antibiotics, most AMPs have amphiphilic structures, and they exhibit antibacterial activity primarily through interactions with the negatively charged bacterial membrane, making it difficult for the bacteria to develop resistance [14]. In addition, their rapid and broad-spectrum antimicrobial activity make them potential therapeutic alternative to antibiotics [15].

In the clinical setting, different antibiotics are often used in combination therapy to broaden the antimicrobial spectra. The main advantage of combination antibiotic therapy is that it can prevent the emergence of MDR bacteria. Antibiotics can exhibit side effects such as diarrhea, rash, nausea, liver damage, and kidney damage; therefore, decreasing drug toxicity through lowering the doses is beneficial [16,17,18]. A few novel AMPs exhibit synergistic effects with known antibiotics against MDR bacteria. (p-BthTX-I)_2_ and LL-37 in combination with florfenicol and thiamphenicol exert antimicrobial activity against *Citrobacter freundii* [19]. Melittin in combination with clindamycin has shown antimicrobial activity against methicillin-resistant *Staphylococcus aureus* [20]. AMPs have also been combined with antibiotics such as T3, T4 with ampicillin and oxacillin [21], WW304 with ciprofloxacin [22], and G3KL with erythromycin and vancomycin [23]. As carbapenem is the most used front-line antibiotic for treating Gram-negative bacterial infections, the accelerating appearance of CRAB seriously threatens global public health [6,7,8]. Antimicrobial activity against MDR-Gram-negative bacteria has been improved through the synergistic effects of SET-M33 [24] or melittin [25] with antibiotics; however, such synergistic combinations with antibiotics to combat CRAB infections remains challenging to develop. AMPs have thus demonstrated some potential regarding combination therapy with conventional antibiotics. Additionally, to overcome drug resistance, AMPs can be easily manipulated to design potent novel AMPs by substituting their amino acid residues. Therefore, the development of AMPs that have synergistic effects with antibiotics against MDR Gram-negative bacteria, and especially clinical CRAB isolates, is important but challenging.

AMPs with improved antimicrobial activities include a series of hybrid peptides that were designed by combining the active regions of two AMPs. For example, cecropin A-magainin 2 (CAMA) and cecropin A-melittin (CAME) hybrid peptides have previously been reported to demonstrate high antimicrobial and antitumor activities [26,27,28]. A hybrid peptide with a broad spectrum of antimicrobial activity (PapMA) was discovered by connecting the N-termini of papiliocin and magainin 2, joined by a proline (Pro) hinge [29]. The structure of PapMA was investigated using nuclear magnetic resonance (NMR) spectroscopy, revealing that it had an N-terminal α-helix from Lys^3^ to Lys^7^ and a C-terminal α-helix from Lys^10^ to Lys^17^, with a Pro^9^ hinge in between. PapMA showed potent antibacterial activity against both Gram-negative and Gram-positive bacteria.

In this study, we aimed to design a novel PapMA analog with increased anti-CRAB activity, while maintaining low cytotoxicity. Its synergistic antibacterial activities against CRAB were then investigated when combined with conventional antibiotics, and the inhibition of biofilm formation was also assessed. In total, six analogs of PapMA were designed by substituting Ala^15^ or Phe^18^ with Ala, Phe, and Trp at the C-terminus. Among the six analogs, we chose PapMA-3 as a candidate for further investigation, as it showed potent anti-CRAB activity with low cytotoxicity. PapMA-3 was found to depolarize CRAB cell membranes, which disrupted biofilm formation and increased susceptibility to the conventional antibiotics. Therefore, in this study, the key mechanism of action underlying this AMP activity was elucidated, suggesting that they are valuable as an adjuvant pharmaceutical to overcome Gram-negative bacterial resistance and represents a good starting point for the development of new antibiotics against CRAB infection.

## 2. Materials and Methods

### 2.1. Peptides and Materials

All peptides were synthesized through N-(9-fluorenyl) methoxycarbonyl solid-phase synthesis and were purified using reversed-phase high-performance liquid chromatography (RP-HPLC, YL9100, Younglin, Korea). Peptide purities were over 95%, as evaluated using an analytical HPLC (C18 column, 4.6 × 250 mm) with two different linear gradients of 0.05% aqueous trifluoroacetic acid (TFA, eluent A) and 0.05% TFA in CH_3_CN (eluent B) at a flow rate of 1.5 mL per min at 25 °C. The molecular masses of the peptides (Table 1) were determined using Axima (Shimadzu Scientific Instruments, Kyoto, Japan) matrix-assisted laser-desorption ionization-time of flight mass spectrometry at the Korea Basic Science Institute (KBSI, Ochang, Korea). The conventional antibiotics (purity over 95%) were purchased as follows: imipenem, meropenem, erythromycin, and rifampin from Sigma-Aldrich (St. Louis, MO, USA), vancomycin from BIO BASIC (Markham, Ontario, Canada), and linezolid from Pharmacia & Upjohn Company (Kalamazoo, MI, USA). 

### 2.2. Antimicrobial Activity 

The Gram-negative bacterial strain *Escherichia coli* (KCTC 1682) and Gram-positive bacterial strain *Staphylococcus aureus* (KCTC 1621) were purchased from the Korean Collection for Type Cultures (Jeongeup, Korea). *Acinetobacter baumannii* (KCCM 40203) were purchased from Korea Culture Center of Microorganisms (Seoul, Korea). Additionally, five carbapenem-resistant *Acinetobacter baumanii* C1–C5 (CRAB C1–C5), which have the OXA-23 gene with carbapenem-resistance were collected from the patients with CRAB bacteremia, who presented symptoms and signs of infection at Korea University Anam Hospital (Seoul, Korea) (IRB registration no. 2020AN0157). The minimum inhibitory concentrations (MIC) of the AMP and antibiotics against the various bacterial strains were assessed using the serial dilution method on Muller–Hinton (MH) media, as described previously [30]. In brief, the peptides at 128 μg·mL^−1^ and antibiotics at 512 μg·mL^−1^ were serially diluted to 1/2 and incubated with a bacterial suspension of 2 × 10^5^ CFU·mL^−1^ in MH media at 37 °C for 16 h. Absorbance at 600 nm was measured using a SpectraMAX microplate reader (Molecular Devices, San Jose, CA, USA).

### 2.3. Peptide-LPS Binding Assay

The capacity of PapMA series peptides to bind with LPS was analyzed using a fluorescent probe, BODIPY-TR cadaverine (BC) (Thermo Fisher Scientific, MA, USA), as described previously [31]. The probe complex was prepared by incubating LPS (50 μg·mL^−^^1^) with BC (5 μg·mL^−^^1^) in a 50 mM Tris buffer (pH 7.4) for 6 h at 25 °C. Varying concentrations of peptides (1–64 μg·mL^−^^1^) were added to a 96-well, dark fluorescence plate and allowed to interact with the LPS–BC complex for 30 min. The fluorescence intensity was recorded at an excitation wavelength of 580 nm and emission wavelength of 620 nm using a SpectraMAX Gemini^TM^ fluorescence microplate reader (Molecular Devices). The %ΔF (A.U.) was calculated using Equation (1):

%ΔF (A.U.) = [(F_obs_ – F_0_) / (F_100_ – F_0_)] × 100 (1)

F_obs_ is the observed fluorescence due to the peptide. F_0_ is the fluorescence without the addition of the peptide. F_100_ is the fluorescence value measured using LL-37, a control peptide with outstanding LPS-neutralizing properties [32].

### 2.4. Membrane Depolarization

The membrane depolarization activity of each AMPs at varying concentrations (1–16 μg·mL^−^^1^) against CRAB C1 intact cells were measured using 3,3′-dipropylthiadicarbocyanine iodide (diSC_3_-5). CRAB C1 was washed two times in washing buffer (5 mM HEPES, 20 mM glucose, pH 7.4), the experiment buffer was changed (5 mM HEPES, 20 mM glucose, 0.1 M KCl, pH 7.4), and diSC_3_-5 dye was added. As a control, 100% depolarization was obtained by treating CRAB C1 with 1% triton X-100 [33]. Spheroplast cells were prepared by the osmotic shock, as previously described [34]. Melittin, which exhibits strong membrane permeabilization, was used for the control treatment at varying concentrations (1–16 μg·mL^−1^). The corresponding fluorescent were measured using RF-6000PC fluorescent spectrophotometer (Shimadzu Scientific Instruments, Kyoto, Japan). 

### 2.5. Time-Dependent Permeabilization of the Outer Membrane 

Time-dependent outer membrane permeabilization activity of PapMA-3 in CRAB C1 intact cells was evaluated using fluorescence-based 1-N-phenylnaphthylamine (NPN). Melittin was used as the control. CRAB C1 cells were washed twice with buffer (5 mM HEPES, 20 mM glucose, pH 7.4) and diluted to OD_600_ = 0.05; 1 μM NPN was added to the cells. Time-dependent NPN uptake was monitored following treatment with PapMA-3 for 30 min. PapMA-3, at varying concentrations (4–32 μg·mL^−1^), was added to the cells, and the fluorescence was measured using the RF-6000PC fluorescent spectrophotometer (Shimadzu Scientific Instruments). 

### 2.6. Cell Cytotoxicity

Human embryonic kidney (HEK)-293 cells, purchased from Korean cell line bank (Seoul, Korea) were cultured in Dulbecco’s modified Eagle’s medium (DMEM) (Welgene, Gyeong-san, Korea) with 10% fetal bovine serum, 1% penicillin-streptomycin at 37 °C in a humidified 5% CO_2_ incubator as described previously [30]. The cytotoxicity of the six PapMA peptides and melittin was determined using WST-8 Cell Proliferation Assay Kit (Biomax Co, Ltd., Seoul, Korea), according to the manufacturer’s instructions. The effects of the most potent peptide, PapMA-3, on mammalian cells were evaluated by measuring the cell activity of HEK-293 cells and human keratinocyte HaCaT cells (Korean cell line bank, Seoul, Korea) after 24 h and 48 h of treatment. The absorbance was measured at 450 nm using a SpectraMAX microplate reader (Molecular Devices).

### 2.7. Stability of PapMA-3 Compared to Melittin in Human Serum

Serum stability of PapMA-3 was assessed by comparing its activity with that of melittin, based on the effects on *E. coli, A. baumannii*, and CRAB C1. MIC was measured in the presence of 50% human serum (Sigma-Aldrich) in the MH medium, in comparison to that of melittin, as described in Section 2.1. Antibacterial activity of PapMA-3 in combination with imipenem was measured against CRAB C1 in the presence of 50% human serum. The treated cells were incubated for 16 h at 37 °C, and the absorbance at 600 nm was measured using a SpectraMAX microplate reader (Molecular Devices). 

### 2.8. Hemolytic Activity

The hemolytic activity of PapMA series peptides was determined against Sheep red blood cells (sRBC) (KisanBio, Seoul, Korea). Fresh sRBC were washed at least three times with phosphate-buffered saline (PBS), followed by centrifugation for 5 min at 1000× *g* at 4 °C. PapMA series peptides (0.25–256 μg·mL^−^^1^) dilute in PBS were incubated with 4% (*v*/*v*) sRBC for 1 h at 37 °C. The contents were then centrifuged at 4 °C for 5 min at 1000× *g*. After transferring the supernatant, absorbance was measured at 405 nm using SpectraMAX microplate reader (Molecular Devices). As a control, 100% hemolysis was obtained by treating sRBC with 1% triton X-100.

### 2.9. Checkerboard Assays

The synergistic effects of AMPs and antibiotics were measured using checkerboard assays [35]. Serial dilutions of PapMA-3 and antibiotics were performed from 1 to ½ of the MIC. Samples were then cross-mixed and cultured in MH medium with 2 × 10^5^ CFU·ml^−^^1^ bacteria. Results were recorded after 16 h of incubation at 37 °C. The fractional inhibitory concentration index (FICI) was calculated according to the European Committee on Antimicrobial Susceptibility (EUCAST) [36]. The FICI was calculated using Equation (2):

(2) $$\mathrm{FICI}=\frac{\left(\mathrm{MIC}\;\mathrm{of}\;\mathrm{PapMA}\text{-}3\;\mathrm{in}\;\mathrm{combination}\right)}{\left(\mathrm{MIC}\;\mathrm{of}\;\mathrm{PapMA}\text{-}3\;\mathrm{alone}\right)}+\frac{\left(\mathrm{MIC}\;\mathrm{of}\;\mathrm{antibiotic}\;\mathrm{in}\;\mathrm{combination}\right)}{\left(\mathrm{MIC}\;\mathrm{of}\;\mathrm{antibiotic}\;\mathrm{alone}\right)}$$

where FICI ≤ 0.5 indicates synergism, 0.5 < FICI < 1 indicates an additive effect, 1 < FICI ≤ 4 represents indifference, and FICI > 4 shows antagonism [37]. 

### 2.10. Time Killing Assay

CRAB C1 cells at 2 × 10^5^ CFU·mL^−^^1^ were incubated with selected concentrations of AMP or antibiotic at 37 °C. At 5, 10, 15, 30, and 40 min and 1, 2, and 4 h, a ten-fold serially diluted suspensions with MH media were spread on an LB agar plate and incubated at 37 °C for 12 h; the number of colonies was counted. 

### 2.11. Scanning Electron Microscope Analysis 

Membrane damage of CRAB C1 was visualized using a field emission scanning electron microscope (FE-SEM), as described previously [38,39], to confirm that the peptides or antibiotics specifically targeted the bacterial membrane. CRAB C1 cells were washed and diluted in PBS to an OD_600_ of 0.2 and incubated with either PapMA-3 or erythromycin or with a combination of 4 μg·mL^−^^1^ PapMA-3 and 128 μg·mL^−^^1^ erythromycin for 15 min or 30 min at 37 °C. The cells were washed using PBS, fixed in 1% osmium tetroxide for 1 h, and dehydrated using a graded ethanol series. After dehydration, ethanol contents in the sample were replaced with varying ratio (2:1, 1:1, 1:2, 0:1 *v*/*v*) of ethanol–isoamyl acetate mixture. The cells were fixed on a glass slide with hexamethyldisilzane, dried under reduced pressure, and platinum-coated; they were visualized using an FE-SEM (SU8020; Hitachi, Tokyo, Japan).

### 2.12. Biofilm Inhibition

Biofilm inhibition activity of PapMA-3 and antibiotics was measured against CRAB C1, as described previously [30]. CRAB C1 cells (2 × 10^5^ CFU·mL^−^^1^) were incubated with PapMA-3 and antibiotics in a tissue-culture well plate in MH medium containing 0.2% glucose for 16 h at 37 °C. The cells were stained with 0.1% (*w*/*v*) crystal violet in 0.25% (*v*/*v*) acetic acid for 1 h at room temperature; the dye complex was dissolved with ethanol. Absorbance at 595 nm was measured using SpectraMAX microplate reader (Molecular Devices) to quantify the biofilm formation.

### 2.13. Isothermal Titration Calorimetry (ITC)

Binding affinity was measured using MicroCal Auto-iTC200 (Malvern Panalytical, Malvern, UK) at the KBSI (Ochang, Korea). Each peptide (0.2 mM; 370 μL) was added to 0.025 mM of LPS (*E. coli* O111:B4, Sigma-Aldrich) in Dulbecco’s phosphate-buffered saline (DPBS, pH 7.0); the injection duration was 2s, the spacing was 150 s, and the temperature was 37 °C. ITC data were analyzed using MicroCal Origin 2020b software (MicroCal origin, MA, USA).

### 2.14. Saturation Transfer Difference (STD)-NMR

STD-NMR experiments were performed at 25 °C on a Bruker 900 MHz spectrometer at KBSI (Ochang, Korea). The STD-NMR spectra were obtained using selective saturation of 15 μM LPS (*E. coli* O111:B4, Sigma-Aldrich, St. Louis, MO, USA) resonances at −4.0 ppm (40 ppm for reference spectra). Peptide was dissolved in 10mM sodium phosphate (pH 6.8, D_2_O) to a concentration of 0.5 mM. For all STD-NMR experiments, a cascade of 40 selective gaussian-shaped pulses of 50 ms duration were used with a total saturation time of 2 s. Difference spectrum was obtained by subtraction of the two spectra (on resonance-off resonance), which shows signals arising from the saturation transfer. 

### 2.15. Statistical Analysis 

Measurements were taken at least three times, and all statistical analyses were performed using the GraphPad Prism software 8.0 for windows (GraphPad Software, CA, USA). The values are expressed as mean ± standard deviation (SD). Statistical significance (*p* < 0.05) was determined using one-way or two-way ANOVA with Dunnett’s test.

## 3. Results

### 3.1. Design of PapMA and Its Analogs

The cationicity and amphiphilicity of antimicrobial peptides are important regarding their binding to bacterial cell membranes via electrostatic interactions with phospholipid head groups, as well as via hydrophobic interactions with membrane lipids [40]. Papiliocin is a 37-residue AMP that was isolated from the swallowtail butterfly (Papilio xuthus) [41]; magainin 2 is a 23-residue AMP isolated from the skin of the African clawed frog (Xenopus laevis) [42,43]. These two peptides are highly cationic, have amphipathic α- helical structures, and have low cytotoxic effects against mammalian cells. Papiliocin has demonstrated high antibacterial activity against Gram-negative bacteria through bacterial membrane disruption, while magainin 2 has displayed high antimicrobial activity against both Gram-negative and Gram-positive bacteria. An 18-residue hybrid peptide (PapMA) was developed by incorporating N-terminal residues 1–8 of papiliocin and N-terminal residues 4–12 of magainin 2, joined by a proline (Pro) hinge [29]. However, the antibacterial activity of PapMA is not potent enough for it to function as a peptide antibiotic.

**Table 1:** Peptides and their physicochemical properties.

Peptides	Sequence	Length	Molecular Weight	Hydrophobicity <H> ^1^	Net Charge ^2^
papiliocin	RWKIFKKIEKVGRNVRDGIIKAGPAVAVVGQAATVVK-NH_2_	37	4002.8	0.300	7
Magainin 2	GIGKFLHSAKKFGKAFVGEIMNS	23	2466.9	0.373	3
PapMA	RWKIFKKIPKFLHSAKKF-NH_2_	18	2302.1	0.394	7
PapMA-2	RWKIFKKIPKFLHSAKKA-NH_2_	18	2225.5	0.312	7
PapMA-3	RWKIFKKIPKFLHSAKKW-NH_2_	18	2340.6	0.419	7
PapMA-4	RWKIFKKIPKFLHSFKKF-NH_2_	18	2377.5	0.476	7
PapMA-5	RWKIFKKIPKFLHSWKKF-NH_2_	18	2416.4	0.502	7
PapMA-6	RWKIFKKIPKFLHSWKKW-NH_2_	18	2455.6	0.527	7

^1^ Hydrophobicity <H> was calculated using http://heliquest.ipmc.cnrs.fr/cgi-bin/ComputParams.py (accessed on 17 August 2021) [44]. Bold letters in sequence represent substituted residues. ^2^ HeliQuest calculates the net charge at pH = 7.4.

**Figure 1:**
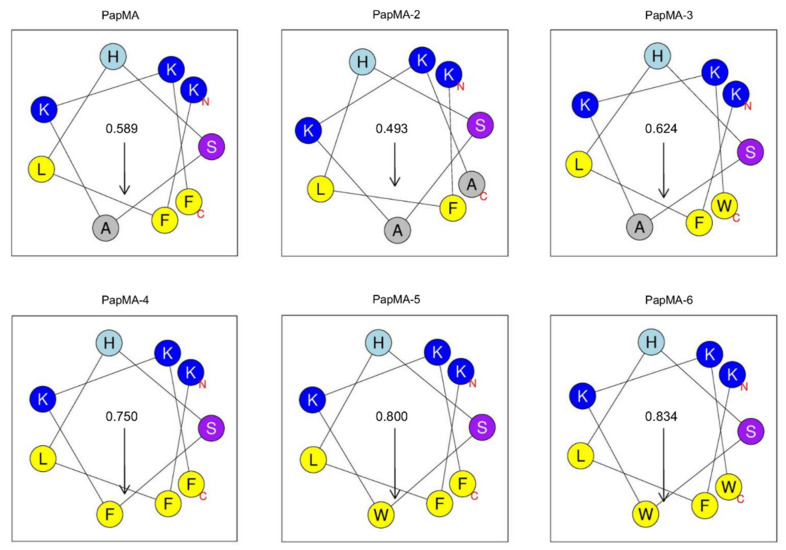
Helical wheel diagram of C-terminal helix from 10th to 18th residue of PapMA and its analogs after Pro hinge generated using HeliQuest at pH 7.4 [44]. Residues at the N-terminus and C-terminus of C-terminal helix are marked as N and C in the figure. Hydrophilic residues are shown in blue. Hydrophobic residues are shown in yellow. Uncharged His is shown in cyan, and Ser is shown in purple. The arrows represent the helical hydrophobic moment.

To improve and optimize the balance between its antibacterial activity and cytotoxicity, here, analogs were designed by changing the hydrophobicity but maintaining the cationicity. A previous study demonstrated that Trp^2^ and Phe^5^ in the N-terminus of papiliocin play important roles in its antibacterial activity. Therefore, new analogs of PapMA were designed here by substituting Ala^15^ or Phe^18^ with Ala, Phe, or Trp at the C-terminus of PapMA to optimize the hydrophobicity and membrane permeabilizing activity, while achieving low cytotoxicity (Table 1). To investigate the role of Phe^18^ at the C-terminus, Phe^18^ was substituted with Ala or Trp (PapMA-2 and PapMA-3, respectively). To increase the hydrophobicity, Ala^15^ was substituted with Phe or Trp (PapMA-4 and PapMA-5, respectively). For PapMA-6, both residues were substituted by Trp. PapMA-2, which had Ala at both positions, exhibited the lowest hydrophobicity (0.312), while PapMa-6, which had Trp at both positions, showed the highest hydrophobicity (0.527; Table 1). The hydrophobic moment of the C-terminal helix was highest in PapMA-6 (0.834), with an order of: PapMA-2 < PapMA < PapMA-3 < PapMA-4 < PapMA-5 < PapMA-6, as shown in Figure 1. The antimicrobial activities and cytotoxicities of peptides were also compared to the parent hybrid peptide, PapMA.

### 3.2. Antimicrobial Activities of PapMA Analogs

Measurement of the minimum inhibitory concentrations (MIC) was conducted to determine the effect of the hydrophobicity of each antimicrobial peptide on its antimicrobial activity. MIC was defined as the minimum concentration that killed more than 99% of bacteria; it was measured against two standard Gram-negative bacteria (*E. coli* and *A. baumanii*), five clinically isolated CRAB (C1–C5), and one Gram-positive bacteria (*S. aureus*). The antimicrobial activities of PapMA and its analogs are listed in Table 2.

In this study, six conventional antibiotics were selected for analysis. Imipenem and meropenem are carbapenem antibiotics that have demonstrated potency against Gram-negative bacteria; they inhibit cell wall synthesis [45]. They have very strong antibacterial activity against *E. coli* and *A. baumanii;* however, their antibacterial activity against carbapenem-resistant CRAB strains is very low. Rifampin has been shown to be potent against *Mycobacterium* and *S. aureus*; it inhibits bacterial deoxyribonucleic acid (DNA)-dependent ribonucleic acid (RNA) polymerase [46]. The antibiotic vancomycin is only potent against Gram-positive bacteria; it inhibits cell wall peptidoglycan synthesis [47]. Erythromycin and linezolid, meanwhile, can bind to 50s ribosome RNA, causing Gram-positive bacterial death through the inhibition of protein synthesis [48]. Compared to PapMA, PapMA-2, which had a lower hydrophobicity due to substitution with Ala, showed a reduced antimicrobial activity. However, PapMA-3, -4, -5, and -6, which exhibited increased hydrophobicities, demonstrated enhanced antimicrobial activities. PapMA and its analogs showed more potent antibacterial activity against Gram-negative bacteria than against Gram-positive bacteria. 

Geometric means (GM) were calculated to compare the relative antimicrobial activities of the analogs against Gram-negative bacteria. The GM values were in the order of PapMA-4 < PapMA-6 < PapMA-5 < PapMA-3 < PapMA < PapMA-2, confirming the improved activities of the analogs compared to PapMA (except for PapMA-2). These results suggest that the increased hydrophobicity had a positive effect on the antimicrobial activity. CRAB C1–C5 are carbapenem-resistant to imipenem and meropenem. In contrast, erythromycin [49], vancomycin [50], and linezolid have shown potent antibacterial activity against Gram-positive bacteria, but much lower antimicrobial activity against Gram-negative bacteria. Next, the antibacterial mechanisms of peptides were investigated.

### 3.3. Antibacterial Mechanisms of PapMA Analogs against CRAB

#### 3.3.1. Binding Assay of LPS-PapMA Analogs

**Figure 2:**
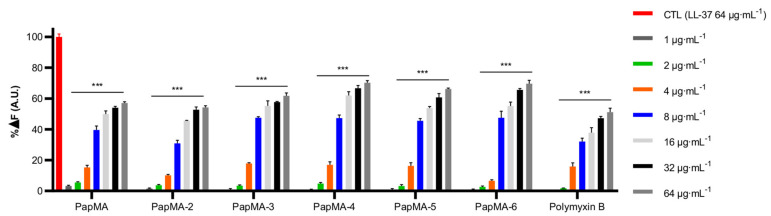
LPS interaction of PapMA analogs. Binding affinity of PapMA derivatives and polymyxin B to LPS based on displacement assays with BODIPY-TR-cadaverine fluorescent dye. Statistical analysis was performed using two-way ANOVA with Dunnett’s comparison test. The values are expressed as the mean ± SEM of three independent experiments and are statistically significant at ***p < 0.001; ns, not significant.

LPS is a major component of the outer membrane of Gram-negative bacteria. It is the permeability barrier of conventional antibiotics, and results in the complication of antibiotic development. Therefore, it is useful to design AMPs that can perturb the bacterial membrane by interacting with LPS. To confirm the antibacterial mechanisms of the developed PapMA analogs against Gram-negative bacteria, the LPS binding mechanisms of the PapMA analogs were investigated using the BC displacement assay (Figure 2). LL-37, which is well-known as the most efficient LPS-neutralizing peptide, was used as a control; its fluorescence intensity at 64 μg·mL^−^^1^ of LL-37 was selected as 100% for comparison. The activity was compared to that of polymyxin B, which is also a well-known LPS-neutralizing peptide [51]. As a result of incubating the LPS-BC complex and the peptides together, all the peptides produced stronger dose-dependent enhancements in fluorescence intensity. All the PapMA analogs showed higher LPS binding interactions than that of polymyxin B. The results showed that LPS interaction increased following the substitution of Ala with Phe or Trp at the C-terminus. Comparing the interactions of PapMA and PapMA-3, LPS interactions increased slightly when Phe^18^ was replaced with Trp. LPS interactions with PapMA-4, -5, and -6 with two aromatic rings at the C-terminus were slightly higher compared to those of PapMA, PapMA-2, and -3. 

#### 3.3.2. Depolarization of PapMA and Its Analogs against CRAB C1

**Figure 3:**
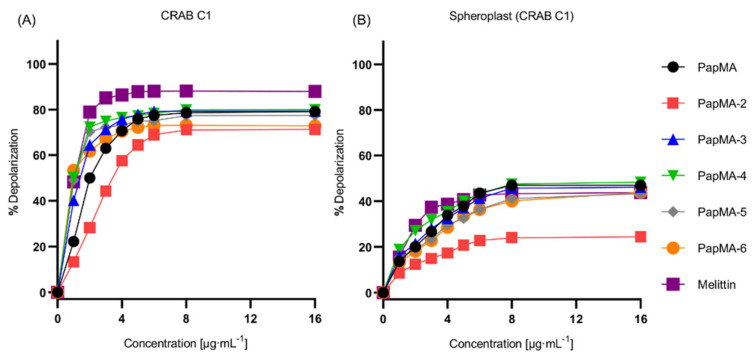
CRAB C1 membrane destruction caused by PapMA analogs. The concentration dependent depolarization of (**A**) intact CRAB C1 and its (**B**) spheroplast induced by PapMA and its analogs, determined using the membrane potential-sensitive fluorescent dye diSC_3_-5. Dye release was monitored by measuring fluorescence, at an excitation wavelength of 654 nm and an emission wavelength of 670 nm.

To elucidate the antibacterial mechanisms of the PapMA analogs on the CRAB C1 membrane, depolarization experiments were performed using intact CRAB C1, as well as CRAB C1 spheroplasts that were created by removing LPS and peptidoglycan; melittin was used as a control. Figure 3A shows that, at a concentration of 8 μg·mL^−^^1^, the depolarization of PapMA analogs and melittin occurred close to the maximum. At 4 μg·mL^−1^ (i.e., half of the concentration of maximum depolarization), depolarization values of 70.7, 57.6, 75.7, 76.5%, 73.2, 70.5, and 86.4% were achieved, respectively. Melittin showed the highest depolarization, while PapMA-2, which had the lowest hydrophobicity, showed the lowest depolarization among all peptides. Interestingly, the PapMA analogs induced bacterial membrane damage even at concentrations much lower than their MICs. When LPS was removed from the CRAB C1 membrane, all peptides displayed approximately 30–40% lower depolarization for CRAB C1 spheroplasts than for the intact membrane (Figure 3B). These results, along with those from the BC displacement assays, indicate that PapMA and its analogs interacted with LPS, major outer membrane component of CRAB, implying that the PapMA peptides targeted and disrupted the outer bacterial membrane more efficiently than the inner membrane. 

### 3.4. Cytotoxicities of PapMA Analogs

**Figure 4:**
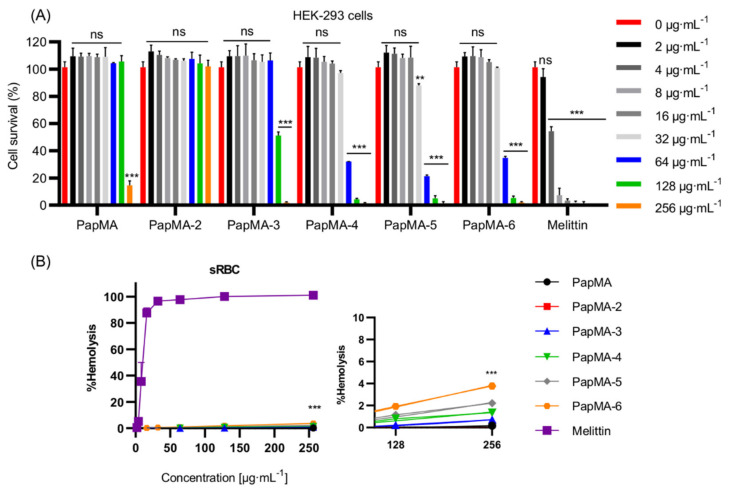
Cytotoxicity of PapMA analogs. (**A**) Cytotoxicity of PapMA and its analogs against HEK-293 cell. The peptide was serially diluted and incubated with cells for 24 h. (**B**) Hemolysis activity of PapMA analogs against sRBC. The peptide was serially diluted and incubated with sRBC for 1 h with melittin as a control. Statistical analysis was performed using two-way ANOVA with Dunnett’s comparison test. The values are expressed as the mean ± SEM of three independent experiments and are statistically significant at ** *p* < 0.01; and *** *p* < 0.001. ns, not significant.

To utilize AMPs as therapeutic agents, they should exhibit low toxicity against mammalian cells [15]. Antibiotics could cause kidney damage; polymyxins, the last-resort antibiotics to treat Gram-negative bacterial infections, have limited use due to its nephrotoxicity [52]. Therefore, to assess the cytotoxicity and to select safe candidates, the cytotoxicities of PapMA and its analogs were investigated against the HEK-293 cell line (Figure 4A). PapMA, PapMA-2, and PapMA-3 showed 100% survival rates at concentrations below 64 μg·mL^−1^, whereas PapMA-4, PapMA-5, and PapMA-6 showed survival rates of 32.1, 21.4, and 34.8%, respectively, at 64 μg·mL^−1^. These results show that cytotoxicity increased proportional to increasing hydrophobicity.

PapMA, PapMA-2, and PapMA-3 showed 100% survival rates at concentrations below 64 μg·mL^−^^1^, whereas PapMA-4, PapMA-5, and PapMA-6 showed survival rates of 32.1, 21.4, and 34.8%, respectively, at 64 μg·mL^−^^1^. These results show that cytotoxicity increased proportional to increasing hydrophobicity.

The hemolytic activity was analyzed against sheep red blood cells (sRBC; Figure 4B). The incubation of sRBC with 256 μg·mL^-1^ for PapMA-4, -5, and -6 induced 1.4, 2.2, and 3.8% hemolysis, respectively. However, PapMA, -2, and -3 caused almost no hemolysis (much lower than 1%). In contrast, melittin exhibited more than 90% hemolysis at 32 μg·mL^−^^1^. These results also confirmed that increases in hydrophobicity led to increases in toxicity, through strong hydrophobic interactions occurred between the aromatic residues of peptides and phospholipids in the mammalian cell membranes (which have higher compositions of neutral phospholipids). Among all six peptides studied, PapMA-3 exhibited the highest bacterial cell selectivity, with a GM of 18.3 and a 100% survival rate at 64 μg·mL^−^^1^ in HEK-293 cells. Even though PapMA-4, -5, and -6 showed potent antibacterial activities, with GMs of 10.3–18.3, they showed severe cytotoxicity (<35% survival rates at 64 μg·mL^−^^1^ in HEK-293 cells). Therefore, PapMA-3 was selected as a candidate therapeutic peptide for further investigation.

### 3.5. Synergistic Effects of PapMA-3 with Antibiotics against Five CRAB 

The appearance of CRAB has accelerated the usage of combination therapy as a new therapeutic approach for its treatment [53,54,55]. As PapMA-3 was selected as a candidate peptide antibiotic, the synergistic effects of PapMA-3 with conventional antibiotics were investigated using checkerboard assays against five clinically isolated CRAB (C1–C5) [35,56]. Regarding the combinations with front-line conventional antibiotics, imipenem and meropenem were used for Gram-negative infections. The synergistic effects of PapMA-3 were also investigated with rifampin, erythromycin, vancomycin, and linezolid, which are well-known antibiotics that are potent against Gram-positive bacteria.

**Figure 5:**
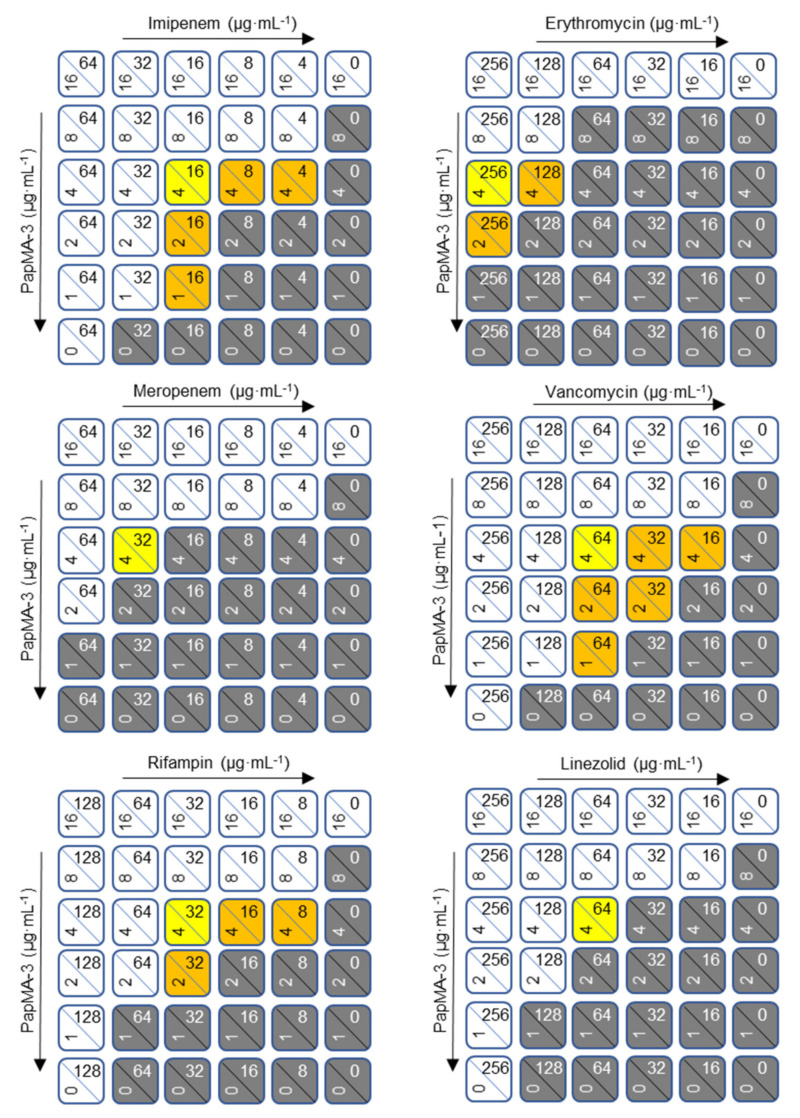
Checkerboard assays of PapMA-3 in combination with six conventional antibiotics against CRAB C1. PapMA-3 and antibiotics were subjected to 1/2 dilution vertically and horizontally from the MIC concentration at the upper left corner. White (0.5 < FICI < 2) indicates a partial synergistic effect, yellow (FICI = 0.5) and orange (FICI < 0.5) indicate a synergistic effect, and gray indicates growth of bacteria. We defined MIC that inhibits completely over 99% of CRAB C1 bacterial growth.

The ability of PapMA-3 to facilitate these antibiotics in penetrating the bacterial membranes of Gram-negative bacteria was investigated. Each peptide was serially diluted to 1/16 from 1 MIC; the experiment was carried out by cross-mixing them. As shown in Figure 5 and Figure S1, the checkerboard assays revealed that PapMA-3 displayed an outstanding synergistic effect with all antibiotics against CRAB C1. PapMA-3 at 4 μg·mL^−1^ (1/4 MIC) displayed synergistic effects towards CRAB C1, exhibiting FICI values lower than 0.50 when combined with all six antibiotics (Table 3). PapMA-3 also showed synergistic effects against CRAB C2 with imipenem (0.38), rifampin (0.25), vancomycin (0.25), and linezolid (0.50; Figure S2). For CRAB C3, PapMA-3 only showed a synergistic effect when combined with rifampin (FICI = 0.38; Figure S3). Among all cases, the combination of PapMA-3 (1 μg·mL^−1^) and rifampin (16 μg·mL^−1^) showed the most effective synergistic effect against CRAB C4, with a FICI value of 0.16 (Figure S4). Figure S5 shows that PapMA-3 demonstrated synergistic effects against CRAB C5 with rifampin, vancomycin, erythromycin, and linezolid; the FICI value was 0.5, respectively. Interestingly, combining PapMA-3 at 2 μg·mL^−1^ (1/8 MIC) and vancomycin at 32 μg·mL^−1^ (1/8 MIC) demonstrated an effective synergistic effect (FICI = 0.25) against both CRAB C1 and C2 (Figure S2). Antibiotics potent for Gram-positive bacteria, such as erythromycin, vancomycin, and linezolid, cannot pass the outer membrane barriers of Gram-negative bacteria; they only demonstrate antibacterial activity against Gram-positive bacteria. Combining PapMA-3 with these antibiotics demonstrated significant antibacterial effects on CRAB, confirming that the interaction of PapMA-3 with the Gram-negative CRAB membrane allowed these antibiotics to penetrate it.

### 3.6. Mechanism of Synergistic Activity of PapMA-3 with Antibiotics against CRAB 

#### 3.6.1. Confirmation of Synergistic Effects between PapMA-3 and Antibiotics through Time Killing Assays 

**Figure 6:**
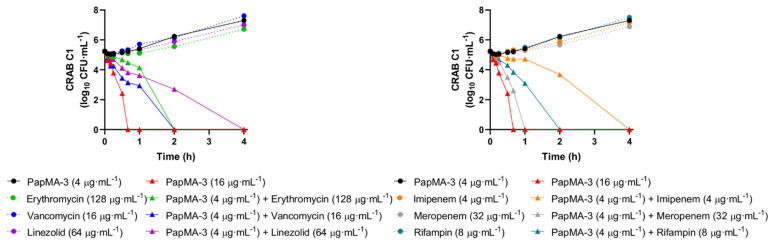
Time-killing curve of PapMA-3 and antibiotics at synergistic concentration against CRAB C1. *Y*-axis indicates CFU in log scale.

Time-killing assays of PapMA-3 alone or in combination with antibiotics against CRAB C1 were performed at those concentrations that showed synergistic effects (FICI < 0.5) in the checkerboard assay. As shown in Figure 6, at the MIC of PapMA-3 (16 μg·mL^−^^1^), peptide treatment completely killed CRAB C1 strains. At a PapMA-3 concentration of 4 μg·mL^−^^1^, for which most combined treatments showed synergistic effects in checkboard assays, the peptide-only treatment did not show any bacterial killing for 4 h. However, when the six antibiotics were incubated at synergistic concentrations in combination with PapMA-3 at 4 μg·mL^−^^1^ (Table 3, Figure S1), meropenem (32 μg·mL^−^^1^) exhibited the most synergistic effect—all bacteria were killed within 1 h. Erythromycin (128 μg·mL^−^^1^), rifampin (8 μg·mL^−^^1^), and vancomycin (16 μg·mL^−^^1^) killed all bacteria within 2 h, while imipenem (4 μg·mL^−^^1^) and linezolid (64 μg·mL^−^^1^) killed all bacteria within 4 h. Therefore, these antibiotics, when combined with PapMA-3 (4 μg·mL^−^^1^), could completely and rapidly kill CRAB C1 in a synergistic manner (Figure 6). 

#### 3.6.2. Visualization of CRAB C1 Membrane Disruption by PapMA-3 in Combination with Antibiotics Using a Field Emission Scanning Electron Microscope (FE-SEM)

**Figure 7:**
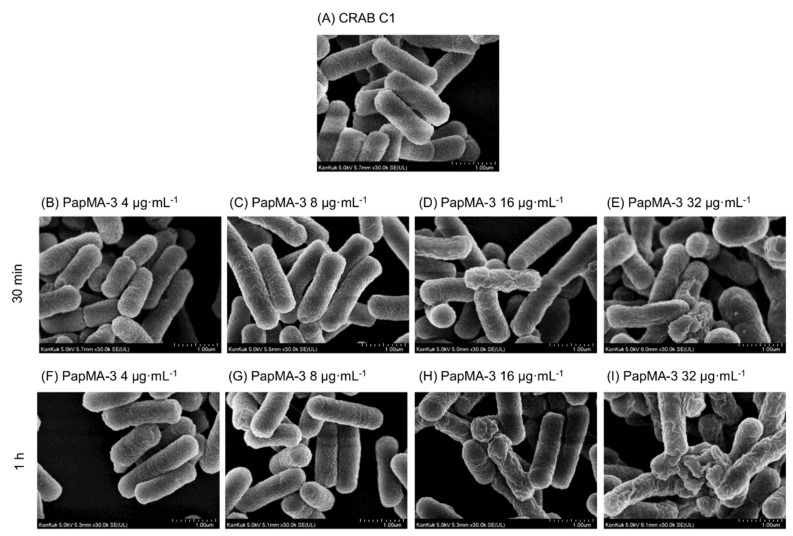
FE-SEM images of CRAB C1 treated with PapMA-3. (**A**) Only CRAB C1. (**B**–**E**) after incubation for 30 min with PapMA-3 at 4 (1/4 MIC), 8 (1/2 MIC), 16 (1 MIC), and 32 μg·mL^−1^ (2 MIC), respectively. (**F**–**I**) same experiments after incubation for 1 h, respectively.

To elucidate the antibacterial mechanism and synergistic effect, the membrane disruption of CRAB by PapMA-3, in combination with antibiotics at concentrations showing synergistic effects, were investigated using an FE-SEM. The changes in the membrane morphology of CRAB C1 were investigated either with PapMA-3 treatment alone or in combination with erythromycin. Figure 7A shows the intact CRAB C1 membrane, revealing that the morphology was maintained at a steady state of membrane integrity, with a smooth surface. As shown in Figure 7B–I, CRAB C1 gradually lost its membrane integrity after 30 min and 1 h as the PapMA-3 concentration increased (4–32 μg·mL^−^^1^). PapMA-3 treatment caused the CRAB membrane surface to become severely roughened and wrinkled, in proportion to the concentration of peptide (Figure 7C–I). Peptide treatment at its MIC (16 μg·mL^−^^1^) after 1 h caused severe damage, supporting the antibacterial mechanism of PapMA-3 via the membrane disruption of CRAB C1.

**Figure 8:**
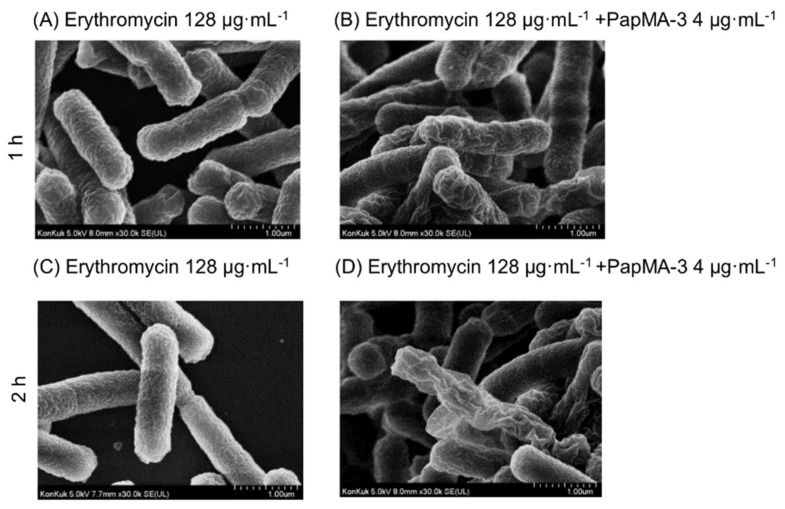
FE-SEM images of CRAB C1 treated with erythromycin and PapMA-3. (**A**) after incubation for 1 h with erythromcyin (128 μg·mL^−1^; synergistic concentration) and (**B**) with erythromycin (128 μg·mL^−1^) + PapMA-3 (4 μg·mL^−1^). (**C**) After incubation for 2 h with erythromcyin (128 μg·mL^−1^; synergistic concentration) and (**D**) with erythromycin (128 μg·mL^−1^) + PapMA-3 (4 μg·mL^−1^).

The membrane integrity of CRAB C1 was not altered by erythromycin itself (128 μg·mL^−^^1^), which was lower than the MIC (Figure 8A,C). However, when CRAB C1 was co-treated with 4 μg·mL^−^^1^ PapMA-3 and 128 μg·mL^−1^ erythromycin, severe membrane disruption was observed at 2 h (Figure 8B,D). Therefore, PapMA-3 helped in the entry of antibiotics through the cell membrane by sufficiently changing the morphology of the membrane. In addition, a combination of PapMA-3 and antibiotics resulted in more efficient membrane damage. These results agree with the result obtained from time killing assay (Figure 6).

To confirm the time-dependent outer membrane permeabilization by PapMA-3, we investigated the time required by PapMA-3 for the membrane permeabilization of outer membrane of CRAB C1 by monitoring NPN uptake at 4, 8, 16, and 32 μg·mL^-1^ of PapMA-3 (Figure S6). Destabilization of the outer membrane by PapMA-3 caused the dye to enter the damaged CRAB C1 membrane, and fluorescence was increased rapidly in a concentration-dependent manner and saturated after 10 min, confirming that PapMA-3 disrupted rapidly outer membrane of CRAB.

### 3.7. Synergistic Effects of PapMA-3 on Biofilm Inhibition

Biofilms confer resistance to bacteria against their environment [57,58]. Biofilm formation can occur on an assortment of surfaces, including living tissues such as wounds and infected skin, as well as on prosthetic implants and various abiotic surfaces [59,60]. The rate of formation of biofilms is high in the case of *A. baumannii*, which is found in urinary catheter, bronchial epithelial cells, as well as abiotic surfaces [61]. Bacterial biofilms confer antibiotic resistance and reduce antibiotic penetrance [62]. 

**Figure 9:**
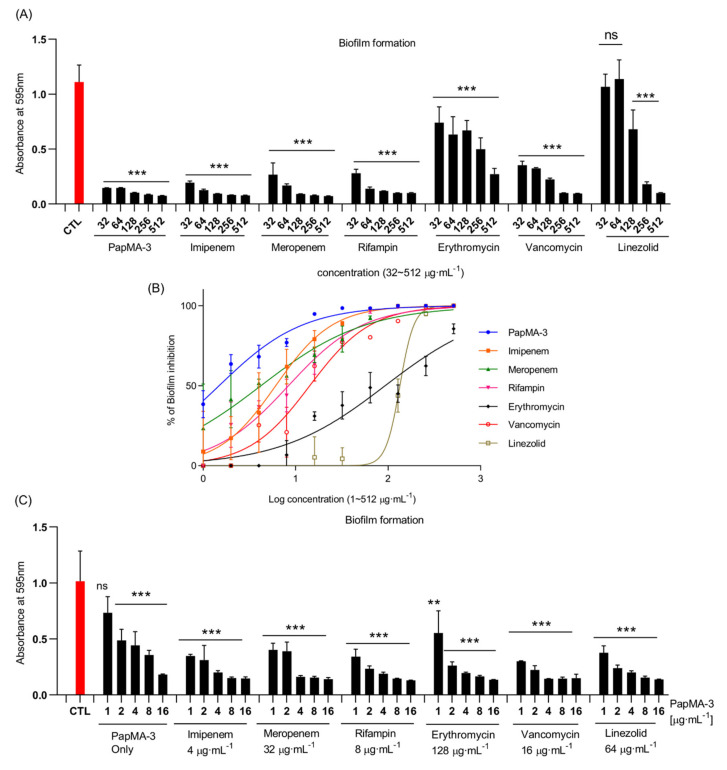
Anti-biofilm activity of PapMA-3 in combination with antibiotics. Biofilms were quantified by staining with crystal violet. (**A**) Absorbance of crystal violet-stained biofilms with treatment of PapMA-3 and antibiotics at a concentration range of 32 to 512 μg·mL^−1^, assessed at 595 nm. (**B**) Confirmation of anti-biofilm activity against CRAB C1 at log scale concentrations (from 1 to 512 μg·mL^−1^) of PapMA-3 and antibiotics, comparative calculation result with CTL of 0% without peptide or antibiotics. (**C**) Synergistic anti-biofilm activities of PapMA-3 and antibiotics against CRAB C1, assessed based on absorbance at 595 nm. CRAB C1 without peptides or antibiotics served as the control (red). Statistical analysis was performed using two-way ANOVA with Dunnett’s comparison test. The values are expressed as the mean ± SEM of three independent experiments and are statistically significant at ** *p* < 0.01 and *** *p* < 0.001. ns, not significant.

Biofilm formation in CRAB C1 was inhibited by PapMA-3 combined with antibiotics (Figure 9). PapMA-3 exhibited a significantly superior biofilm inhibition activity against CRAB C1 compared with that of the other tested antibiotics, in a concentration-dependent manner. Biofilm inhibition was quantified by measuring the absorbance at 595 nm of the crystal violet-stained biofilms. Absorbance treated with 32 μg·mL of PapMA-3, imipenem, meropenem, rifampin, erythromycin, vancomycin, and linezolid were 0.15, 0.19, 0.27, 0.28, 0.74, 0.35, and 1.07, respectively (Figure 9A). The absorbance of biofilm formed by CRAB C1 without peptide or antibiotics served as control was 1.11. The percentage of biofilm inhibition caused by these antibiotics at 32 μg·mL^−1^ was 98.5, 88.9, 79.2, 77.5, 37.7, 77.2, and 4.4%, respectively, compared to the control (Figure 9B). 

However, co-treatments comprising 4 μg·mL^−^^1^ PapMA-3 with antibiotics (4 μg·mL^−^^1^ imipenem, 32 μg·mL^−^^1^ meropenem, 8 μg·mL^−^^1^ rifampin, 128 μg·mL^−^^1^ erythromycin, 16 μg·mL^−^^1^ vancomycin, or 64 μg·mL^−^^1^ linezolid) showed synergistic effects (Table 3); the absorbance at 595 nm for these co-treatments were less than 0.20. Thus, it can be concluded that combining PapMA-3 with antibiotics can deliver superior therapeutic effects compared to using antibiotics alone, regarding the inhibition of biofilm formation. This occurred due to the effect of PapMA-3 on inducing the permeabilization of the bacterial membrane (Figure 9C).

### 3.8. Stability and Effects of PapMA-3 on Mammalian Cells Compared to That of Melittin 

#### 3.8.1. Stability of PapMA-3 Compared to That of Melittin in the Presence of Human Serum

High stability is necessary for the in vivo efficacy of peptides. Peptides are degraded by proteases and other components in the serum; therefore, we measured the stability of PapMA-3 alone or in combination with imipenem in human serum to confirm its potential as an AMP candidate [25]. The antibacterial activity of PapMA-3 was reduced four-fold in the presence of 50% human serum in MH media (Table 4), while melittin lost antibacterial activity considerably. Checkerboard assays revealed that 4 μg·mL^−^^1^ PapMA-3 displayed an outstanding synergistic effect with 4 μg·mL^−^^1^ imipenem, exhibiting FICI value of 0.31 against CRAB C1 (Table 3). PapMA-3 in combination with 16 μg·mL^−^^1^ imipenem retained its antibacterial activity at 16 μg·mL^−^^1^, even in the presence of 50% serum (Table 4). Even though PapMA-3 contains all L-amino acids in the sequence, these results ascertain the potential of PapMA-3 for therapeutic applications and combinational therapy can compensate the problems caused by the instability of peptide antibiotics in the serum.

#### 3.8.2. Effects of PapMA-3 Compared to That of Melittin on Mammalian Cells

**Figure 10:**
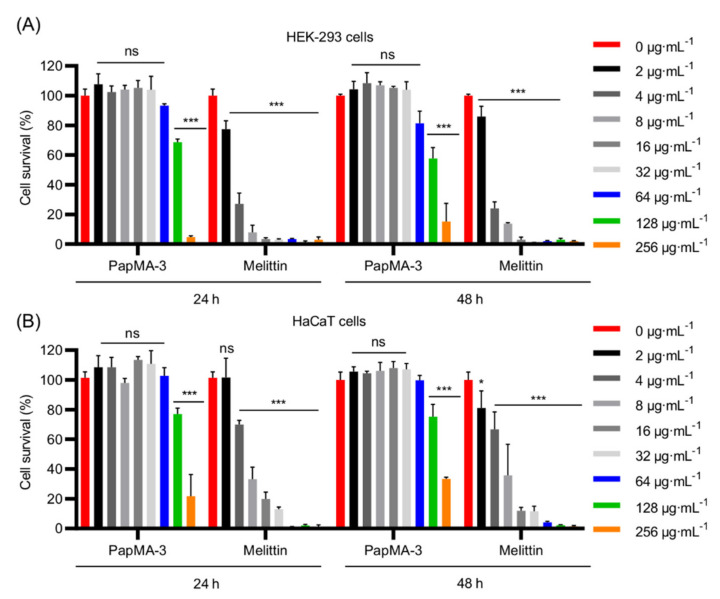
Cytotoxicity of PapMA-3 (**A**) Cytotoxicity of PapMA-3 against HEK-293 cells at 24 h and 48 h. (**B**) Cytotoxicity of PapMA-3 against HaCaT cell at 24 h and 48 h. Statistical analysis was performed using two-way ANOVA with Dunnett’s comparison test. The values are expressed as the mean ± SEM of three independent experiments and are statistically significant at * *p* < 0.05; *** *p* < 0.001; ns, not significant.

We investigated the effect of PapMA-3 on the mammalian cells, HEK-293, and HaCaT for 48 h to evaluate its cytotoxicity (Figure 10). Cell activities were monitored at 24 h and 48 h following the peptide treatment. At 32 μg·mL^−^^1^, the cell proliferation and viability remained unaltered at 24 h and 48 h compared to that of the blank control. Even at 64 μg·mL^−^^1^, viability was reduced to less than 20% at 24 h and at 48 h compared to the control. In contrast, treatment with melittin caused severe toxicity and significantly reduced viability at 24 h and 48 h, even at its MIC. Therefore, PapMA-3 could be a potent antibiotic peptide.

### 3.9. Binding Interactions of PapMA-3 with LPS as Studied by STD-NMR Spectroscopy and ITC

**Figure 11:**
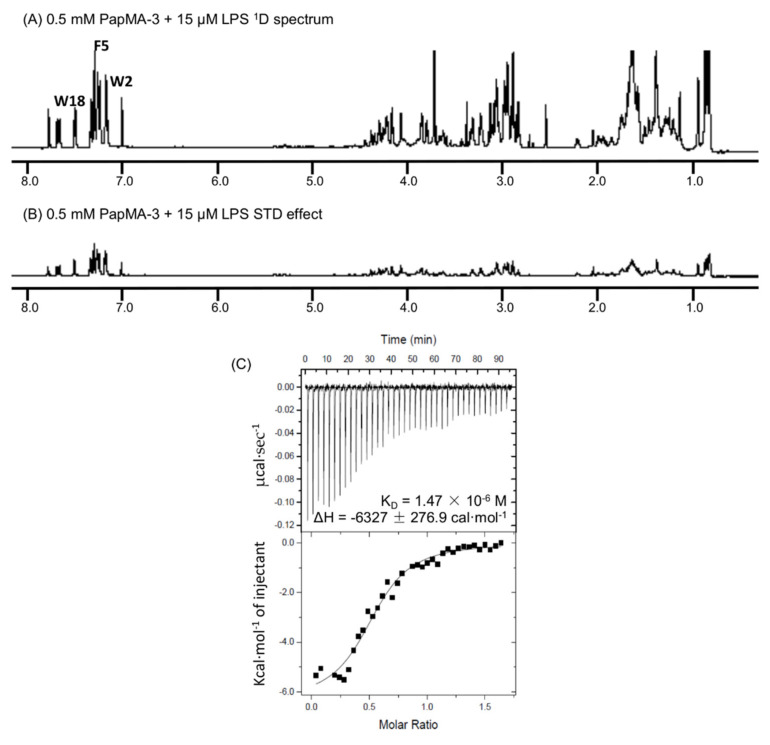
Binding interaction of PapMA-3 with LPS. Saturation transfer difference (STD) NMR analysis of interaction between PapMA-3 and LPS in D_2_O at 298 K. (**A**) ^1^D ^1^H NMR spectra of 0.5 mM PapMA-3 plus 15 μM LPS (sample A), (**B**) STD NMR spectrum obtained on sample A at 298 K. (**C**) Isothermal titration calorimetry (ITC) measurement showing the binding affinity of 0.2 mM PapMA-3 to 0.025 mM LPS from E. coli O55:B5.

STD NMR experiments were conducted to clarify the antibacterial mechanism of PapMA-3. To determine which residues in PapMA-3 were the most favorable to LPS binding, they were compared to ^1^D ^1^H NMR spectra of PapMA-2 (with Ala^18^) and PapMA-3 (with Trp^18^); a previously obtained spectrum of PapMA was also used [63]. The STD effect was determined using the spectral differences; it primarily constituted resonances belonging to peptide protons bound to LPS. Significant STD effects were identified in the aromatic ring region for Trp^2^, Phe^5^, and Trp^18^ (in the region of 7.8–7.4 ppm). This confirmed that all aromatic residues at both the N- and C-termini had direct molecular interactions with LPS (Figure 11A,B). Furthermore, protons in aliphatic regions also showed an STD effect with LPS, confirming that PapMA-3 enacted antibacterial activity via strong LPS interactions, resulting in disruption of CRAB bacterial membrane. 

The binding affinity of PapMA-3 to LPS was further investigated using ITC, revealing that an exothermic process with strong electrostatic interactions occurred between PapMA-3 and LPS, with a binding affinity of 1.47 × 10−6 M at 37 °C (Figure 11C). The STD-NMR spectroscopy and ITC results together confirmed that PapMA-3 exhibited antibacterial activity via its strong interaction with LPS; thereby, it can enhance the membrane permeability of conventional antibiotics.

## 4. Discussion 

The discovery and advancement of antibiotics initially seemed to have effectively combated diseases caused by bacterial infections; however, the overuse of antibiotics has led to the emergence of MDR bacterial strains. As a countermeasure against resistant strains, multiple antibiotics can be used in combination. In clinical settings, this strategy is advantageous, as it can broaden the target spectra against pathogens and prevent the development of drug resistance by reducing the amounts of antibiotics used. Furthermore, combination therapy can decrease the toxicity by allowing lower doses of the combined harmful drugs to be used. Combination therapies for antibiotics that have recently been approved by the US Food and Drug Administration (FDA) include ceftolozane/tazobactam, ceftazidime/avibactam, and meropenem/vaborbactam; furthermore, imipenem/relebactam and aztreonam/avibactam remain under clinical research [64]. 

Many studies have explored the combination of AMPs and antibiotics. The emergence of resistant strains to carbapenem, which is an important antibiotic against Gram-negative bacteria, has intensified the need for new alternatives for the treatment of CRAB pathogens classified as critical MDR bacteria by WHO [2]. However, few studies have synergistically investigated the combined effects of AMPs and antibiotics against Gram-negative bacteria, due to complications posed by the bacterial membranes. For example, Ω76 has been studied regarding its synergistic effects on CRAB; an FICI value of 0.56 was obtained with colistin [54], demonstrating a partial synergistic effect via a synergistic mechanism that enhanced the membrane permeability of antibiotics. The combination of melittin and doripenem has also shown a very good synergistic combination, achieving a FICI value of <0.1 against CRAB, whereas melittin was found not to exhibit a synergistic effect with doxycycline and colistin [25]. However, the severe toxicity of melittin can limit the clinical application. SET-M33 has showed synergistic effects with aztreonam, meropenem, rifampin, and tobramycin against CRAB strain [24]. 

In clinical trials, combinations of colistin and conventional antibiotics are mainly used to treat MDR Gram-negative bacteria [65,66]. Although colistin itself has excellent antibacterial activities, its high nephrotoxicity is a factor that limits its use alone; the appearance of colistin-resistant bacteria also limits its usage. For example, a randomized clinical trial of colistin in combination with meropenem is currently ongoing in Europe and the United States (ClinicalTrials.gov IDs NCT01732250 and NCT01597973) [67]. Additionally, clinical trials of colistin and rifampin in Korea have confirmed the presence of a partial synergistic effect (NCT03622918) [68]. However, in these studies, the combination treatments have not been shown to be superior to colistin monotherapy, as no similar or significant differences have been obtained [65,66].

Mechanisms of antibiotic resistance in bacteria include thickening the membrane to lower the permeability of antibiotics, creating an efflux pump to re-release antibiotics, modifying the target of antibiotics, and inactivating antibiotics by decomposing them [69]. Carbapenem antibiotics are members of β-lactam antibiotics, which inhibit synthesis of bacterial cell wall by binding to penicillin-binding proteins. Furthermore, carbapenem resistance mechanisms have been described in *A. baumannii*, including the alteration or loss of outer membrane proteins and efflux modifications [70]. Among many carbapenem-hydrolyzing oxacillinase-encoding genes, OXA-23 is widespread in Korea, and the number of antibiotics available to treat CRAB are decreasing [71]. The present study aimed to find an efficient treatment method for CRAB infections using combinational therapy of the newly designed PapMA-3 and six conventional antibiotics, which included antibiotics that are potent against Gram-negative or Gram-positive bacteria. PapMA-3-antibiotic combinations were assessed against five clinical isolates, OXA-23-producting CRAB (C1–C5), and the underlying mechanism was explored. 

To facilitate the uptake of antibiotics through the LPS outer membrane, PapMA-3 showed strong interactions with LPS and depolarized the CRAB outer membrane, while demonstrating low cytotoxicity. Its binding interactions with LPS were investigated using BC displacement assays, ITC, and STD-NMR experiments, confirming that membrane permeabilization via strong binding to LPS was the major antibacterial mechanism. PapMA-3 showed a superior BC displacement to a well-known LPS-neutralizing peptide, polymyxin B, by binding the core part of LPS, lipid A [72,73]. The therapeutic potential of PapMA-3 against CRAB was examined in combination with imipenem and meropenem, which are effective against Gram-negative bacteria. Furthermore, PapMA-3 was also combined with four antibiotics that have demonstrated antibacterial activity against Gram-positive bacteria. Outstanding synergistic effects (FICI < 0.5) between PapMA-3 and all six antibiotics were confirmed against both CRAB C1 and C4 clinical isolates. In particular, combining PapMA-3 with rifampin, vancomycin, and erythromycin achieved efficient synergistic effects against CRAB C4, with FICI values of <0.25, implying that PapMA-3 disrupted the membrane integrity of CRAB, allowing the antibiotics that are effective against Gram-positive bacteria to enter and reach their intracellular targets in the CRAB cells. Additionally, PapMA-3 might help imipenem and meropenem to overcome the CRAB membrane; however, underlying mechanism is not yet clearly understood.

Biofilm formation by MDR bacteria aids antibiotic resistance; it needs to be overcome due to its effects in causing pneumonia, meningitis, bacteremia, wounds, and soft-tissue infections [74]. PapMA-3 itself was able to suppress biofilm formation at its MIC, but it was also able to suppress sufficiently biofilm formation at lower concentrations when combined with antibiotics. This implies that combinational therapies constituting PapMA-3 and conventional antibiotics could be applied clinically. FE-SEM images suggested that PapMA-3 destabilized the morphology of the bacterial membrane even at concentrations below the MIC. Importantly, the CRAB membrane was destroyed when PapMA-3 was applied in combination with erythromycin, which alone are only effective against Gram-positive bacteria. Time killing assays suggested that the combinations of PapMA-3 with meropenem or erythromycin completely and rapidly killed CRAB C1 (within 1 h). Therefore, this combinational therapy could be applied to the enable the usage of potent antibiotics against both Gram-negative and Gram-positive bacteria by facilitating membrane permeability.

However, several problems persist that need to be addressed in future studies. First, the resistance to protease needs to be improved in our peptides by introducing D-amino acids, non-natural amino acids, or cyclization [75,76,77]. Additionally, these synergistic effects need to be confirmed by in vivo animal experiments before this combinational therapy can be applied clinically. Additionally, underlying mechanisms for synergistic effect on combination therapy should be investigated further in our future studies.

## 5. Conclusions

In this study, PapMA-3, a novel peptide, was designed and demonstrated potent anti-microbial activity against CRAB without notable cytotoxicity against mammalian cells. PapMA-3 was shown to target the outer bacterial membrane of CRAB via a strong interaction with LPS. At synergistic concentrations, PapMA-3 was found to cause the partial depolarization of the CRAB membrane, which changed the membrane morphology sufficiently to allow the antibiotics to penetrate intracellularly. This synergistic usage of PapMA-3 with well-known antibiotics resulted in the killing of CRAB and the inhibition of their biofilm formation. This was even achieved when the antibiotics used had previously only demonstrated potency against Gram-positive bacteria. This study may provide insights regarding the development of alternative therapies that utilize novel peptide antibiotics in combination with classical antibiotics to treat CRAB infections.

## 6. Patents

Patent applications for these peptides have been registered in Korea (101875057). These peptides have given rise to patent number PCT/KR2017/006650, and patent applications have been completed in United State (SOP114552US) and China (201780039278.1).

## Figures and Tables

**Table 2:** Antibacterial activities of antimicrobial peptides and antibiotics against microorganisms.

Peptides	Minimum Inhibitory Concentration (μg·mL^−1^)								
	Gram-Negative Bacteria								Gram-Positive Bacteria
	*E. coli*	*A. baumanii*	CRAB C1	CRAB C2	CRAB C3	CRAB C4	CRAB C5	GM ^1^	*S.aureus*
PapMA	32	32	32	32	16	64	32	34	64
PapMA-2	64	128	128	>128	64	>128	128	146	>128
PapMA-3	16	16	16	16	16	32	16	18	32
PapMA-4	16	8	8	8	8	16	8	10	32
PapMA-5	16	16	16	16	16	32	16	18	32
PapMA-6	16	8	8	8	8	16	16	11	32
melittin	8	16	16	16	16	8	16	14	16
**Antibiotics**									
Imipenem	0.25	0.25	64	64	64	64	64	46	1
Meropenem	0.25	0.25	128	64	64	128	64	64	1
Rifampin	2	4	128	64	128	128	256	101	1
Erythromycin	16	32	>512	512	512	>512	>512	592	0.25
Vancomycin	128	256	256	256	256	512	256	274	0.5
Linezolid	256	256	256	256	256	512	512	329	1

^1^ The geometric means (GMs) are the mean minimum inhibitory concentration (MIC) values of Gram-negative bacterial strains. The GMs were assumed to be 256 μg·mL^−^^1^ for MIC > 128 μg·mL^−^^1^ and 1024 μg·mL^−^^1^ for MIC > 512 μg·mL^−^^1^.

**Table 3:** FICI for the synergistic effect of PapMA-3 in combination with antibiotics against CRABs.

PapMA-3 with Antibiotics		MIC (μg·mL^−1^)		MIC in Combination (μg·mL^−1^)		
	Strain	PapMA-3	Antibiotics	PapMA-3	Antibiotics	FICI ^#^
PapMA-3 + Imipenem	CRAB C1	16	64	4.0	4.0	0.31 *
	CRAB C2	16	64	4.0	8.0	0.38
	CRAB C3	16	64	8.0	1.0	0.52
	CRAB C4	32	64	8.0	8.0	0.38
	CRAB C5	16	64	8.0	16	0.75
PapMA-3 + Meropenem	CRAB C1	16	128	4.0	32	0.50
	CRAB C2	16	64	8.0	32	1.00
	CRAB C3	16	64	8.0	16	0.75
	CRAB C4	32	128	8.0	32	0.50
	CRAB C5	16	64	8.0	16	0.75
PapMA-3 + Rifampin	CRAB C1	16	128	4.0	8.0	0.31
	CRAB C2	16	64	2.0	8.0	0.25
	CRAB C3	16	128	4.0	16	0.38
	CRAB C4	32	128	2.0(8.0)	8.0(4.0)	0.13(0.27)
	CRAB C5	16	256	4.0	64	0.50
PapMA-3 + Erythromycin	CRAB C1	16	>512	4.0	128	0.38
	CRAB C2	16	512	8.0	128	0.75
	CRAB C3	16	512	8.0	64	0.63
	CRAB C4	32	>512	4.0(8.0)	64(16)	0.19(0.27)
	CRAB C5	16	>512	4.0	256	0.50
PapMA-3 + Vancomycin	CRAB C1	16	256	2.0(4.0)	32(16)	0.25(0.31)
	CRAB C2	16	256	2.0	32	0.25
	CRAB C3	16	256	8.0	4.0	0.52
	CRAB C4	32	512	4.0(8.0)	64(16)	0.25(0.28)
	CRAB C5	16	256	4.0	64	0.50
PapMA-3 + Linezolid	CRAB C1	16	256	4.0	64	0.50
	CRAB C2	16	256	4.0	64	0.50
	CRAB C3	16	256	8.0	128	1.00
	CRAB C4	32	512	8.0	64	0.38
	CRAB C5	16	512	4.0	128	0.50

^#^ The fractional inhibitory concentration index (FICI) was calculated according to Equation (1). If the MIC value was not obtained at the highest concentration measured due to poor antibacterial activity, the FICI was considered to be twice the value of the measurement limit. * Combinations that showed synergistic effects are shaded in gray. Where there were multiple sets of combinations with low FICI values, they are listed in parentheses.

**Table 4:** Measurement of serum stability of PapMA-3 and melittin against *E.coli*, *A.baumannii*, and CRAB C1.

Microorganisms	Minimum Inhibitory Concentration (μg·mL^−1^)							
	PapMA-3		Melittin		Imipenem		PapMA-3 + Imipenem	
	MH Media	+ Serum (50%)	MH Media	+ Serum (50%)	MH Media	+ Serum (50%)	MH Media	+ Serum (50%)
*E. coli*	16	64	8	256				
*A. baumannii*	16	64	16	128				
CRAB C1	16	64	16	256	64	64	4 + 4	16 + 16

## Data Availability

Not applicable.

## Supplementary Materials

The following are available online at https://www.mdpi.com/article/10.3390/pharmaceutics13111800/s1, Figure S1: PapMA-3 and antibiotic checkboard assay results, showing fractional inhibitory concentration index (FICI) calculated against CRAB C1 according to Equation (1), Figure S2: PapMA-3 and antibiotic checkboard assay results, showing FICI calculated against CRAB C2., Figure S3: PapMA-3 and antibiotic checkboard assay results, showing FICI calculated against CRAB C3, Figure S4: PapMA-3 and antibiotic checkboard assay results, showing FICI calculated against CRAB C4, Figure S5: PapMA-3 and antibiotic checkboard assay results, showing FICI calculated against CRAB C5.
