# Supplementary material for: Development of Novel Peptides for the Antimicrobial Combination Therapy against Carbapenem-Resistant Acinetobacter baumannii Infection
Source: Pharmaceutics. 2021 Oct 27;13(11):1800. doi: 10.3390/pharmaceutics13111800 (PMC8619914; doi:10.3390/pharmaceutics13111800)
Supplement: Supplementary file 1 [file pharmaceutics-13-01800-s001.zip › pharmaceutics-1382767-supplementary.pdf]

pharmaceutics MDPI

Supplementary Materials: Development of Novel Peptides for the Antimicrobial Combination Therapy Against Carbapenem-resistant *Acinetobacter Baumannii* Infection

Joonhyeok Choi, Ahjin Jang, Young Kyung Yoon and Yangmee Kim

100~99%	
99~90%	
89~50%	
49~20%	
19~0%	

FICI		CRAB C1						
		Imipenem (μg·mL^{-1})						
		64	32	16	8	4	2	0
PapMA-3 (μg·mL^{-1})	16	2.00	1.50	1.25	1.13	1.06	1.03	1.00
	8	1.50	1.00	0.75	0.63	0.56	0.53	
	4	1.25	0.75	0.50	0.38	0.31	0.28	
	2	1.13	0.63	0.38	0.25	0.19		
	1	1.06	0.56	0.31	0.19			
	0	1.00						

FICI		CRAB C1						
		Erythromycin (μg·mL^{-1})						
		512	256	128	64	32	16	0
PapMA-3 (μg·mL^{-1})	16	1.50	1.25	1.13	1.06	1.03	1.02	1.00
	8	1.00	0.75	0.63	0.56	0.53	0.52	
	4	0.75	0.50	0.38	0.31	0.28	0.27	
	2	0.63	0.38	0.25	0.19			
	1	0.56	0.31	0.19	0.13			
	0							

FICI		CRAB C1						
		Meropenem (μg·mL^{-1})						
		64	32	16	8	4	2	0
PapMA-3 (μg·mL^{-1})	16	1.50	1.25	1.13	1.06	1.03	1.02	1.00
	8	1.00	0.75	0.63	0.56	0.53	0.52	
	4	0.75	0.50	0.38				
	2	0.63	0.38					
	1	0.56						
	0							

FICI		CRAB C1						
		Vancomycin (μg·mL^{-1})						
		256	128	64	32	16	8	0
PapMA-3 (μg·mL^{-1})	16	2.00	1.50	1.25	1.13	1.06	1.02	1.00
	8	1.50	1.00	0.75	0.63	0.56	0.53	
	4	1.25	0.75	0.50	0.38	0.31		
	2	1.13	0.63	0.38	0.25	0.19		
	1	1.06	0.56	0.31	0.19	0.13		
	0	1.00						

FICI		CRAB C1						
		Rifampin (μg·mL^{-1})						
		128	64	32	16	8	4	0
PapMA-3 (μg·mL^{-1})	16	2.00	1.50	1.25	1.13	1.06	1.03	1.00
	8	1.50	1.00	0.75	0.63	0.56	0.53	
	4	1.25	0.75	0.50	0.38	0.31		
	2	1.13	0.63	0.38	0.25			
	1	1.06	0.56	0.31	0.19			
	0	1.00						

		CRAB C1						
		Linezolid (μg·mL^{-1})						
		256	128	64	32	16	8	0
PapMA-3 (μg·mL^{-1})	16	2.00	1.50	1.25	1.13	1.06	1.03	1.00
	8	1.50	1.00	0.75	0.63	0.56	0.53	
	4	1.25	0.75	0.50	0.38	0.31		
	2	1.13	0.63	0.38	0.25			
	1	1.06	0.56	0.31	0.19			
	0	1.00						

Supplementary Figure S1. PapMA-3 and antibiotic checkboard assay results, showing fractional inhibitory concentration index (FICI) calculated against CRAB C1 according to Equation 1. White (0.5 < FICI < 2) indicates a partial synergistic effect, yellow (FICI = 0.5) and orange (0.25 ≤ FICI < 0.5) indicate a synergistic effect. We defined MIC as the minimum concentration that killed more than 99% of bacteria. In contrast, the box marked in black indicates that inhibition of CRAB C1 growth was less than 99%.

FICI	CRAB C2						
	Imipenem (μg·mL^{-1})						
PapMA-3 (μg·mL^{-1})	64	32	16	8	4	2	0
16	2.00	1.50	1.25	1.13	1.06	1.03	1.00
8	1.50	1.00	0.75	0.63	0.56	0.53	
4	1.25	0.75	0.50	0.38			
2	1.13	0.63	0.38	0.25			
1	1.06	0.56	0.31	0.19			
0	1.00						

FICI	CRAB C2						
	Erythromycin (μg·mL^{-1})						
PapMA-3 (μg·mL^{-1})	512	256	128	64	32	16	0
16	2.00	1.50	1.25	1.13	1.06	1.03	1.00
8	1.50	1.00	0.75	0.63	0.56	0.53	
4	1.25	0.75	0.50	0.38	0.31		
2	1.13	0.63	0.38	0.25			
1	1.06	0.56	0.31	0.19			
0	1.00						

FICI	CRAB C2						
	Meropenem (μg·mL^{-1})						
PapMA-3 (μg·mL^{-1})	64	32	16	8	4	2	0
16	2.00	1.50	1.25	1.06	1.03	1.02	1.00
8	1.50	1.00	0.75	0.63	0.56	0.53	
4	1.25	0.75					
2	1.13	0.63					
1	1.06						
0	1.00						

FICI	CRAB C2						
	Vancomycin (μg·mL^{-1})						
PapMA-3 (μg·mL^{-1})	256	128	64	32	16	8	0
16	2.00	1.50	1.25	1.13	1.06	1.03	1.00
8	1.50	1.00	0.75	0.63	0.56	0.53	
4	1.25	0.75	0.50	0.38	0.31		
2	1.13	0.63	0.38	0.25	0.19	0.16	
1	1.06	0.56	0.31	0.19	0.13		
0	1.00						

FICI	CRAB C2						
	Rifampin (μg·mL^{-1})						
PapMA-3 (μg·mL^{-1})	64	32	16	8	4	2	0
16	2.00	1.50	1.25	1.13	1.06	1.03	1.00
8	1.50	1.00	0.75	0.63	0.56	0.53	
4	1.25	0.75	0.50	0.38	0.31	0.28	
2	1.13	0.63	0.38	0.25	0.19	0.16	
1	1.06	0.56	0.31	0.19	0.13	0.10	
0	1.00						

	CRAB C2						
	Linezolid (μg·mL^{-1})						
PapMA-3 (μg·mL^{-1})	256	128	64	32	16	8	0
16	2.00	1.50	1.25	1.13	1.06	1.03	1.00
8	1.50	1.00	0.75	0.63	0.56		
4	1.25	0.75	0.50	0.38			
2	1.13	0.63	0.38	0.25			
1	1.06	0.56	0.31	0.19			
0	1.00						

Supplementary Figure S2. PapMA-3 and antibiotic checkboard assay results, showing FICI calculated against CRAB C2.

FICI		CRAB C3						
		Imipenem (µg·mL⁻¹)						
		64	32	16	8	4	1	0
PapMA-3 (µg·mL⁻¹)	16	2.00	1.50	1.25	1.13	1.06	1.02	1.00
	8	1.50	1.00	0.75	0.63	0.56	0.52	
	4	1.25	0.75	0.50	0.38	0.31	0.27	
	2	1.13	0.63	0.38		0.19		
	1	1.06	0.56	0.31				
	0	1.00						

FICI		CRAB C3						
		Erythromycin (µg·mL⁻¹)						
		512	256	128	64	32	16	0
PapMA-3 (µg·mL⁻¹)	16	2.00	1.50	1.25	1.13	1.06	1.03	1.00
	8	1.50	1.00	0.75	0.63	0.56	0.53	
	4	1.25	0.75	0.50	0.38	0.31		
	2	1.13	0.63	0.38	0.25			
	1	1.06	0.56	0.31				
	0	1.00						

FICI		CRAB C3						
		Meropenem (µg·mL⁻¹)						
		64	32	16	8	4	2	0
PapMA-3 (µg·mL⁻¹)	16	2.00	1.50	1.25	1.06	1.03	1.02	1.00
	8	1.50	1.00	0.75	0.63	0.53	0.52	
	4	1.25	0.75	0.50				
	2	1.13	0.63	0.38				
	1	1.06	0.56					
	0	1.00						

FICI		CRAB C3						
		Vancomycin (µg·mL⁻¹)						
		256	128	64	32	8	4	0
PapMA-3 (µg·mL⁻¹)	16	2.00	1.50	1.25	1.13	1.03	1.02	1.00
	8	1.50	1.00	0.75	0.63	0.53	0.52	
	4	1.25	0.75	0.50	0.38			
	2	1.13	0.63	0.38				
	1	1.06	0.56	0.31				
	0	1.00						

FICI		CRAB C3						
		Rifampin (µg·mL⁻¹)						
		128	64	32	16	8	4	0
PapMA-3 (µg·mL⁻¹)	16	2.00	1.50	1.25	1.13	1.06	1.03	1.00
	8	1.50	1.00	0.75	0.63	0.56	0.53	
	4	1.25	0.75	0.50	0.38	0.31	0.28	
	2	1.13	0.63	0.38	0.25	0.19	0.16	
	1	1.06	0.56	0.31	0.19	0.13	0.10	
	0	1.00						

		CRAB C3						
		Linezolid (µg·mL⁻¹)						
		256	128	64	32	16	8	0
PapMA-3 (µg·mL⁻¹)	16	2.00	1.50	1.25	1.13	1.06	1.03	1.00
	8	1.50	1.00	0.75	0.63	0.56	0.53	
	4	1.25	0.75	0.50				
	2	1.13	0.50					
	1	1.06	0.38	0.19				
	0	1.00						

Supplementary Figure S3. PapMA-3 and antibiotic checkboard assay results, showing FICI calculated against CRAB C3.

FICI		CRAB C4						
		Imipenem (μg·mL^{-1})						
		64	32	16	8	2	1	0
PapMA-3 (μg·mL^{-1})	32	2.00	1.50	1.25	1.13	1.03	1.02	1.00
	16	1.50	1.00	0.75	0.63	0.53	0.52	
	8	1.25	0.75	0.50	0.38	0.28	0.27	
	4	1.13	0.63	0.38	0.25	0.16	0.14	
	2	1.06	0.56	0.31	0.19	0.10	0.08	
	0	1.00						

FICI		CRAB C4						
		Erythromycin (μg·mL^{-1})						
		512	128	64	32	16	4	0
PapMA-3 (μg·mL^{-1})	32	1.50	1.13	1.06	1.03	1.02	1.01	1.00
	16	1.00	0.63	0.56	0.53	0.52	0.51	
	8	0.75	0.38	0.31	0.28	0.27	0.26	
	4	0.63	0.25	0.19	0.16	0.14	0.13	
	2	0.56	0.19	0.13	0.10	0.08	0.07	
	0							

FICI		CRAB C4						
		Meropenem (μg·mL^{-1})						
		128	64	32	16	8	4	0
PapMA-3 (μg·mL^{-1})	32	2.00	1.50	1.25	1.13	1.06	1.03	1.00
	16	1.50	1.00	0.75	0.63	0.63	0.53	
	8	1.25	0.75	0.50	0.38	0.38	0.28	
	4	1.13	0.63	0.38	0.25		0.16	
	2	1.06	0.56	0.31	0.19	0.19		
	0	1.00						

FICI		CRAB C4						
		Vancomycin (μg·mL^{-1})						
		512	256	128	64	32	16	0
PapMA-3 (μg·mL^{-1})	32	2.00	1.50	1.25	1.13	1.06	1.03	1.00
	16	1.50	1.00	0.75	0.63	0.56	0.53	
	8	1.25	0.75	0.50	0.38	0.31	0.28	
	4	1.13	0.63	0.38	0.25	0.19	0.16	
	2	1.06	0.56	0.31	0.19	0.13	0.10	
	0	1.00						

FICI		CRAB C4						
		Rifampin (μg·mL^{-1})						
		128	32	16	8	4	2	0
PapMA-3 (μg·mL^{-1})	32	2.00	1.25	1.13	1.06	1.03	1.02	1.00
	16	1.50	0.75	0.63	0.56	0.53	0.52	
	8	1.25	0.50	0.38	0.31	0.28	0.27	
	4	1.13	0.38	0.25	0.19	0.16	0.15	
	2	1.06	0.31	0.19	0.13	0.10	0.08	
	0	1.00						

		CRAB C4						
		Linezolid (μg·mL^{-1})						
		256	128	64	32	16	8	0
PapMA-3 (μg·mL^{-1})	32	1.50	1.25	1.13	1.06	1.03	1.02	1.00
	16	1.00	0.75	0.63	0.56	0.53	0.52	
	8	0.75	0.50	0.38	0.31	0.28	0.27	
	4	0.63	0.38	0.25	0.19	0.16		
	2	0.56	0.31	0.19			0.08	
	0							

Supplementary Figure S4. PapMA-3 and antibiotic checkboard assay results, showing FICI calculated against CRAB C4.

FICI		CRAB C5						
		Imipenem (µg·mL^{-1})						
		64	32	16	8	4	2	0
PapMA-3 (µg·mL^{-1})	16	2.00	1.50	1.25	1.13	1.06	1.03	1.00
	8	1.50	1.00	0.75	0.63	0.56	0.53	
	4	1.25	0.75	0.50	0.38	0.31	0.28	
	2	1.13	0.63	0.38	0.25	0.19	0.16	
	1	1.06	0.56	0.31	0.19	0.13	0.10	
	0	1.00						

FICI		CRAB C5						
		Erythromycin (µg·mL^{-1})						
		512	256	128	64	32	16	0
PapMA-3 (µg·mL^{-1})	16	1.50	1.25	1.13	1.06	1.03	1.02	1.00
	8	1.00	0.75	0.63	0.56	0.53	0.52	
	4	0.75	0.50	0.38	0.31	0.28	0.27	
	2	0.63	0.38	0.25	0.19	0.16	0.14	
	1	0.56	0.31	0.19	0.13	0.10	0.08	
	0							

FICI		CRAB C5						
		Meropenem (µg·mL^{-1})						
		64	32	16	8	4	2	0
PapMA-3 (µg·mL^{-1})	16	2.00	1.50	1.25	1.06	1.03	1.02	1.00
	8	1.50	1.00	0.75	0.63	0.56	0.53	
	4	1.25	0.75	0.50				
	2	1.13	0.63	0.38				
	1	1.06	0.56					
	0	1.00						

FICI		CRAB C5						
		Vancomycin (µg·mL^{-1})						
		256	128	64	32	16	8	0
PapMA-3 (µg·mL^{-1})	16	2.00	1.50	1.25	1.13	1.03	1.02	1.00
	8	1.50	1.00	0.75	0.63	0.56	0.53	
	4	1.25	0.75	0.50	0.38	0.31	0.28	
	2	1.13	0.63	0.38	0.25	0.19	0.16	
	1	1.06	0.56	0.31	0.19			
	0	1.00						

FICI		CRAB C5						
		Rifampin (µg·mL^{-1})						
		256	128	64	32	16	8	0
PapMA-3 (µg·mL^{-1})	16	2.00	1.50	1.25	1.13	1.03	1.02	1.00
	8	1.50	1.00	0.75	0.63	0.56	0.53	
	4	1.25	0.75	0.50	0.38	0.31	0.28	
	2	1.13	0.63	0.38	0.25	0.19	0.16	
	1	1.06	0.56	0.31	0.19	0.13	0.10	
	0	1.00						

		CRAB C5						
		Linezolid (µg·mL^{-1})						
		256	128	64	32	16	8	0
PapMA-3 (µg·mL^{-1})	16	1.50	1.25	1.13	1.06	1.03	1.02	1.00
	8	1.00	0.75	0.63	0.56	0.53	0.52	
	4	0.75	0.50	0.38	0.31	0.28	0.27	
	2	0.63	0.38	0.25	0.19	0.16	0.14	
	1	0.56	0.31	0.19	0.13	0.10	0.08	
	0							

Supplementary Figure S5. PapMA-3 and antibiotic checkboard assay results, showing FICI calculated against CRAB C5.

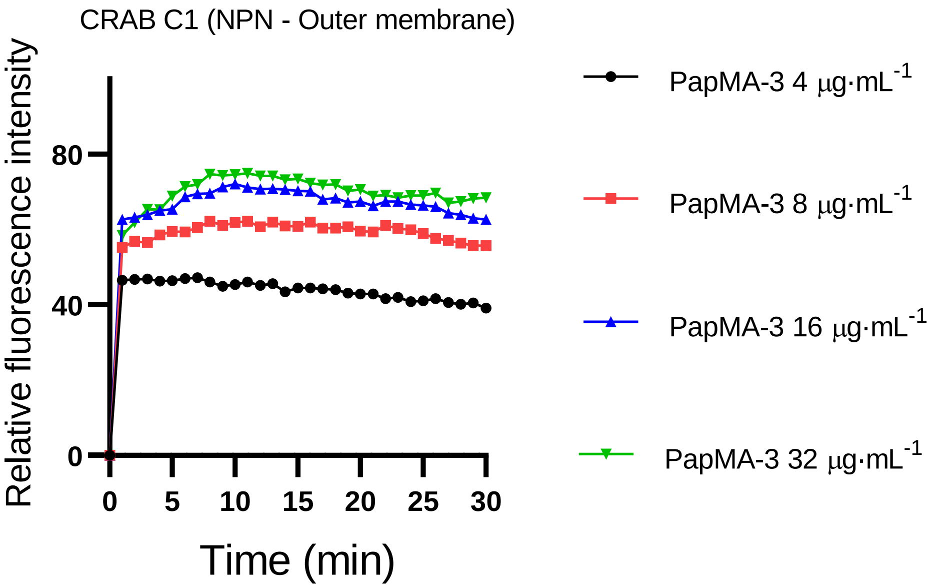

Supplementary Figure S6. Time-dependent outer membrane permeabilization of CRAB C1 by PapMA-3. Increase of NPN fluorescence intensity was monitored (excitation wavelength of 350 nm and an emission wavelength of 420 nm).
